# Instability-negative pressure loss model of gas drainage borehole and prevention technique: A case study

**DOI:** 10.1371/journal.pone.0242719

**Published:** 2020-11-23

**Authors:** Qingjie Qi, Xinlei Jia, Xinhua Zhou, Youxin Zhao

**Affiliations:** 1 School of Safety Science and Engineering, Liaoning Technical University, Fuxin, Liaoning, China; 2 Key Laboratory of Mine Thermodynamic Disasters and Control of Ministry of Education, Fuxin, Liaoning, China; 3 School of Civil Engineering, Liaoning Technical University, Fuxin, Liaoning, China; China University of Mining and Technology, CHINA

## Abstract

The internal collapse of deep seam drainage borehole and negative pressure loss represents a serious technical problem affecting gas drainage. To address this problem a creep model of coal around borehole was established based on the plastic softening characteristics of coal. The final collapse time of the borehole was determined and used to derive the three stages of the borehole collapse process. The model of negative pressure loss in drainage borehole was established according to the theory of fluid dynamics, the model of methane gas flow and the creep model of the coal around the borehole. The relationship between the negative pressure loss of drainage and the change of borehole aperture was derived, thereby revealing the main influencing factors of the negative pressure loss in the borehole. A drainage technique named “Full-hole deep screen mesh pipe” was introduced and tested to prevent the collapse of borehole and reduce the negative pressure loss. The result shows that after the borehole was drilled, the borehole wall was affected by the complex stress of the deep coal seam, the coal surrounding the borehole collapsed or presented the characteristics of creep extrusion towards the borehole. The “Full-hole deep screen mesh pipe drainage technology” could effectively control the collapse as well as the deformation of the borehole and reduced the negative pressure loss. Compared with the traditional drainage technology, the methane gas drainage concentration was increased by 101% and the gas flow was increased by 97% when the methane gas was drained for 90 days, the gas drainage efficiency increased significantly.

## 1. Introduction

Gas drainage is the fundamental method to reduce the pressure of methane gas and its content in coal seams, and it is also a direct way to solve gas disasters and realize the transformation of gas resources into treasure [1–6]. In recent years, with the rapid development of coal mining technology and equipment, the mining intensity has constantly been increasing, and mining level has continuously extended deeper. When coal mining enters the deep part, it is in the complex mechanical environment of “three high and one disturbance” (High geostress, high geothermal, high karst water pressure and strong mining disturbance) [7–10]. Under this condition, during the coal seam gas drainage process, the borehole diameter will change due to the creep characteristics of the coal itself, and the borehole may even collapse in some cases [11, 12]. Danesh N. [13] indicated that the creep deformation of coal reduces its permeability and seriously affects the gas drainage of coal. In addition, the deformation of gas drainage borehole makes the frictional drag and the local resistance larger, which induces the negative pressure of gas drainage in the borehole to decrease rapidly and eventually reach zero. Therefore, it is of great theoretical and engineering significance to study the deformation of gas drainage borehole and the rule of negative pressure loss in gas drainage boreholes to determine the appropriate drainage technology and improve the gas drainage efficiency.

With regards to the recent studies related to creep characteristics of coal and rock mass, Wang and Wang [14] established a new nonlinear creep model, describing the three stages of nonlinear creep of coal and rock. Jiang et al. [15] modified and extended the Nishihara model to obtain a phase of quadratic accelerating creep, and gave the axial creep strain equation under the condition of triaxial creep test. Li et al. [16] applied elastic and viscoelastic theories and the Maxwell model to obtain the analytical solution of coal pillar deformation. They also predicted the displacement of coal pillar and roadway through its solution. Liu et al. [17] analyzed the stability of the borehole and proposed that the instability of the borehole was caused by the influence of confining pressure unloading. Zhang et al. [18] indicated that the stability of gas drainage boreholes was related to the complex stress of the roadway. Through numerical simulations, they concluded that the size of the cohesive force of the roadway was the main factor affecting the deformation of the borehole. Xi, Zhao and Wang et al. [19–22] studied critical conditions of borehole deformation instability and established a mechanical model of borehole deformation.

Several studies have been examined the mechanism of negative pressure loss of gas drainage. Ji [23] considered that the loss of methane gas flow was mainly caused by frictional drag. The differential equation of two-dimensional methane gas flow was established, and the negative pressure loss of borehole extraction was simulated. According to the occurrence and flow law of gas in coal seam, Hu [24] established a numerical model of gas flow in coal seam during negative pressure drainage and derived the distribution law equation of negative pressure in one-way flow of coal seam gas drainage borehole. Liu [25] considered the radial flow of coal seam gas in the borehole, and obtained the relationship between the negative pressure loss and the gas flow of the borehole, which was in the form of a power exponential relationship. Through field experiments, Li and Wang et al. [26, 27] systematically studied the change of negative pressure in the borehole with the length of the borehole. They established correlation between the negative pressure of gas drainage with the change of borehole length through fitting.

In summary, most previous studies have given detailed answers on the relationship between creep deformation of roadway, stability of borehole and roadway stress. However, as per our knowledge, there exists few studies that focused on instability collapse of borehole. In the studies on negative pressure loss of gas drainage, most only considered the change of the negative pressure of the orifice and the influence of the flow of gas in the coal seam on the negative pressure loss. However, the analysis of the drainage negative pressure loss in the borehole based on the creep of coal around the borehole has not been performed. In this study, the creep model of gas drainage borehole was established based on the creep of surrounding rock. Also, based on the fluid dynamics theory and the creep model of borehole, the model of negative pressure loss in gas drainage was derived. Based on the actual parameters of a coal mine, the surrounding rock stress of the roadway was divided, and the causes of instability collapse and negative pressure loss in the drainage borehole were thoroughly analyzed. Finally, the prevention technology for the phenomenon of borehole collapse and negative pressure loss was proposed, and the effect of the prevention technology was investigated, thereby providing an effective technical way for improving the gas drainage effect of borehole in deep coal seam and reducing the pressure loss.

## 2. Development of the mathematical model

### 2.1 Creep model of gas drainage borehole

**Fig 1:**
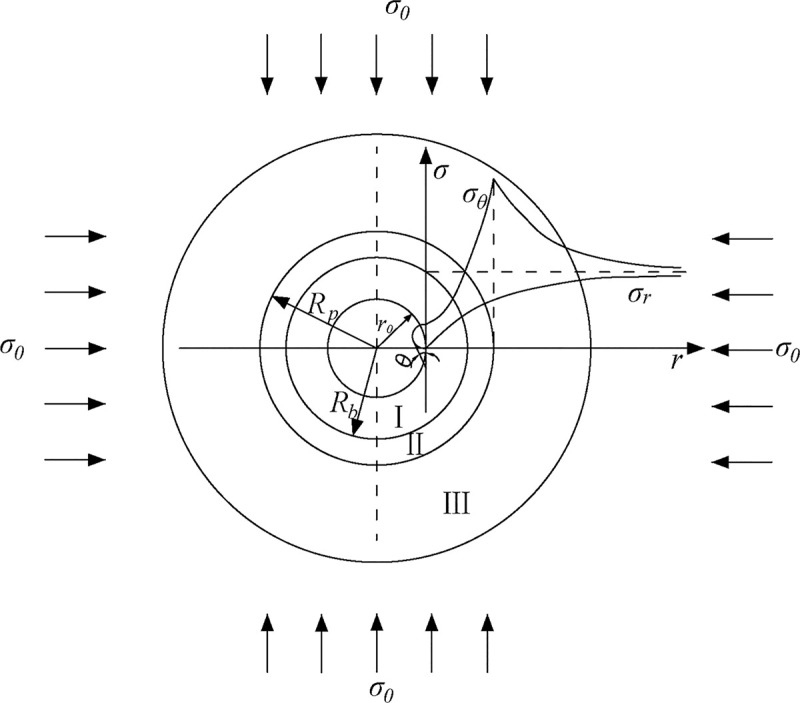
Stress state of the coal around the borehole. *σ*_0_— Pressure of the borehole surrounding the rock, *φ*— Internal friction angle, *r*_0_— Initial radius of the borehole, *R*_*b*_, *R*_*p*_— Radius of the crushing zone and plastic zone, *σ*_*r*_, *σ*_*θ*_ *—*Radial stress and tangential stress, Ⅰ—Crushing zone, Ⅱ—Plastic zone, Ⅲ—Elastic zone.

After the gas drainage borehole is drilled in the deep coal seam, the stress of the coal rock around the borehole will be redistributed to form the crushing zone, plastic softening zone and viscoelastic zone, as shown in Fig 1, which is caused by the plastic softening, dilatancy and rheological properties of the coal and rock mass. In order to further investigate the stress and displacement changes of the coal around the borehole and simplify the model, the following assumptions should be made: ① The coal around the borehole is homogeneous and isotropic [28]. ② The borehole is in the hydrostatic stress field and the borehole is infinitely long, which is a axisymmetric plane strain problem; ③ The coal around the borehole satisfies the Poynting-Thomson model [29]. ④ The coal around the borehole yields to the Mohr-Coulomb strength criterion [30]. ⑤ The coal in the plastic softening zone and the failure zone of the borehole satisfies the flow law of volume expansion [31].

The aforementioned assumptions induce the following equations:

$$The\;static\;equilibrium\;condition:\;\frac{d\sigma_{r}}{dr}+\frac{\sigma_{r}-\sigma_{\theta }}{r}=0,\;\varepsilon_{r}=\frac{du}{dr},\;\varepsilon_{\theta }=\frac{u}{r}$$ (1)

Where *σ*_*r*_, *σ*_*θ*_ is the radial stress and the tangential stress borne by the coal, respectively, *ε*_*r*_, *ε*_*θ*_ is the radial strain and tangential strain of the coal, respectively, *u* is the radial displacement.

$$The\;physical\;property\;equation\;of\;Poynting‐Thomson\;model:\;\sigma +\eta \frac{\partial }{\partial t}\sigma =2G_{\infty }\varepsilon +2G_{0}\eta \frac{\partial }{\partial t}\varepsilon$$ (2)

Where *σ* is the stress of the coal, *ε* is the coal strain, *η* is the relaxation time, *G*_0_, *G*_∞_ is the instantaneous shear modulus and the long-term shear modulus of the coal, respectively.

$$The\;Mohr‐Coulomb\;yield\;criterion:\;\sigma_{\theta }=\frac{1+\sin\varphi }{1-\sin\varphi }\sigma_{r}+\sigma_{c}$$ (3)

Where *φ* is the internal friction angle of coal, *σ*_*c*_ is the compressive strength of coal.

$$The\;flow\;rule\;of\;dilatancy\;of\;coal:\;\Delta \varepsilon_{r}+\eta \Delta \varepsilon_{\theta }=0$$ (4)

Where Δ*ε*_*r*_, Δ*ε*_*θ*_ is the radial and tangential strain increment of the coal, respectively, *η* is the dilatancy coefficient.

The manifestation of drainage borehole is as follows: ① When the pressure on coal exceeds its compressive strength, the coal enters the plastic softening stage. Hence, the strength of the coal gradually attenuates until it reaches a residual strength with the further development of the creep deformation of the coal. ② The volume of the coal expands during plastic softening and deformation, which indicates the occurrence of a dilatancy phenomenon in the coal; ③ The coal has obvious rheological properties, which means that creep deformation occurs over time. The deformation process of the coal around the borehole can be simplified into three stages: viscoelasticity, plastic softening, and crushing, as shown in Fig 1.

Using the model of surrounding rock fracture range and displacement [22, 31] and the linear viscoelastic-plastic softening model of rock progressive failure [32], the elastic zone, the plastic zone and the crushing zone are analyzed, according to the above constitutive equation and yield criterion. The solutions are as follows:

a. The elastic zone

Since the volume of the elastic zone is unchanged and the displacement at the junction of the elastoplastic zone is equal, then:

$$\varepsilon_{r}^{e}+\varepsilon_{\theta }^{e}=\frac{du}{dr}+\frac{u}{r}=0$$ (5)

$$\left\{ \begin{array}{l} \sigma_{r}=\sigma_{0}-\frac{MR_{p}^{2}\left(t\right)}{r^{2}}\\ \sigma_{\theta }=\sigma_{0}+\frac{MR_{p}^{2}\left(t\right)}{r^{2}} \end{array}\right.$$ (6)

Combining Eqs (1), (2), (5) and (6) can be derived as follows:

$$u^{e}=\frac{A\left(t\right)R_{p}^{2}\left(t\right)}{r}$$ (7)

Where *A*(*t*) is a function of time such that:

$$A\left(t\right)=\frac{M}{2}\left\{\frac{1}{G_{\infty }}\left[1-e^{\left(-\frac{t}{\eta_{ret}}\right)}\right]+\frac{1}{G_{0}}e^{\left(-\frac{t}{\eta_{ret}}\right)}\right\}$$ (8)

$$M=\frac{\sigma_{0}\left(K_{p}-1\right)+\sigma_{c}}{K_{p}+1}$$ (9)

Where *u*^*e*^ refers to the displacement of the elastic zone of the borehole, *A*(*t*) is a function of time *t*, *σ*_0_ is the pressure of the rock surrounding the borehole, *σ*_*c*_ is the compressive strength of the coal, *R*_*p*_ is the radius of the plastic softening zone, *η*_*ret*_ is the delay time of the coal rheology around the borehole, *r* is the radius of the borehole, *K*_*p*_ is the pressure coefficient such that *K*_*p*_ = (1+sin*φ*)/(1−sin*φ*), where *φ* is the internal friction angle of coal.

b. The plastic zone

The radial strain and the tangential strain in the plastic zone can be expressed as follows:

$$\left\{ \begin{array}{l} \varepsilon_{r}^{p}={\left(\varepsilon_{r}^{p}\right)}_{r=R_{p}\left(t\right)}+\Delta \varepsilon_{r}^{p}\\ \varepsilon_{\theta }^{p}={\left(\varepsilon_{\theta }^{p}\right)}_{r=R_{p}\left(t\right)}+\Delta \varepsilon_{\theta }^{p} \end{array}\right.$$ (10)

At the junction of the elastoplastic zone:

$${\left(u^{p}\right)}_{r=R_{p}\left(t\right)}=A\left(t\right)R_{p}\left(t\right)$$ (11)

Combining Eqs (1), (4), (10) and (11), one can derive that:

$$u^{p}=\frac{2A\left(t\right)r}{1+\eta_{1}}{\left(\frac{R_{p}\left(t\right)}{r}\right)}^{1+\eta_{1}}+\frac{\eta_{1}-1}{\eta_{1}+1}A\left(t\right)r$$ (12)

Where *u*^*p*^ is the displacement of the plastic zone of the borehole, *η*_1_ is the dilatancy coefficient of coal in the plastic softening zone.

c. The crushing zone

The radial and tangential strains in the crushing zone can be written as follows:

$$\left\{ \begin{array}{l} \varepsilon_{r}^{b}={\left(\varepsilon_{r}^{p}\right)}_{r=R_{b}\left(t\right)}+\Delta \varepsilon_{r}^{b}\\ \varepsilon_{\theta }^{b}={\left(\varepsilon_{\theta }^{p}\right)}_{r=R_{b}\left(t\right)}+\Delta \varepsilon_{\theta }^{b} \end{array}\right.$$ (13)

Combining Eqs (1), (4), and (13), the displacement at the junction of the plastic zone and the crushing zone is equal. It can thus be derived that:

$$u^{b}=2A\left(t\right)r\left\{\left\{\frac{1}{1+\eta_{1}}+\frac{1}{1+\eta_{2}}\left[{\frac{R_{b}\left(t\right)}{r}}^{1+\eta_{2}}-1\right]\right\}{\left(\frac{R_{p}\left(t\right)}{R_{b}\left(t\right)}\right)}^{1+\eta_{1}}+\frac{\eta_{1}-1}{2\left(\eta_{1}+1\right)}\right\}$$ (14)

Where *u*^*b*^ is the displacement of the crushing zone of the borehole, *R*_*b*_ is the radius of the crushing zone, *η*_2_ is the dilatancy coefficient of the coal in the crushing zone.

d. Solving formula of borehole aperture

In the plastic softening zone, the coal strength $\sigma_{c}^{p}$ is a function of strain. According to the most commonly used stress-strain model, $\sigma_{c}^{p}$ can be expressed as follows:

$$\sigma_{c}^{p}=\sigma_{c}-M_{c}\left[\varepsilon_{\theta }^{p}-{\left(\varepsilon_{\theta }^{e}\right)}_{r=R_{p}\left(t\right)}\right]$$ (15)

In the crushing zone, it is considered that the residual strength of the coal is fixed, then there is:

$$\sigma_{\theta }^{b}=K_{p}\sigma_{r}^{b}+\sigma_{c}^{*}$$ (16)

When the coal around the borehole is in a crushing state, there is *r* = *R*_*b*_(*t*), $\sigma_{c}^{p}=\sigma_{c}^{*}$ at the junction of the plastic zone and the crushing zone. Thus, it can be obtained that:

$$R_{p}\left(t\right)=NR_{b}\left(t\right)$$ (17)

$$N={\left[1+\frac{\left(1+\eta_{1}\right)\left(\sigma_{c}-\sigma_{c}^{*}\right)}{2A\left(t\right)M_{c}}\right]}^{\frac{1}{1+\eta_{1}}}$$ (18)

At the junction of the crushing zone and the plastic zone, the radial stresses of the coal around the borehole is equal, which means *r* = *r*_0_, $\sigma_{c}^{b}=\sigma_{0}$. Hence, the radius of the crushing zone is:

$$R_{b}\left(t\right)=r_{0}{\left\{\left\{\frac{2}{K_{p}+1}\left[\sigma_{0}+\frac{\sigma_{c}}{K_{p}-1}+\frac{\left(K_{p}+1\right)M_{c}A\left(t\right)}{\left(K_{p}-1\right)\left(K_{p}+\eta_{1}\right)}\right]•N^{1-K_{p}}+\frac{2M_{c}A\left(t\right)}{1+\eta_{1}}\left[\frac{N^{1+\eta_{1}}}{K_{p}+\eta_{1}}-\frac{1}{K_{p}-1}\right]-\frac{\sigma_{c}}{K_{p}-1}\right\}•\frac{K_{p}-1}{\sigma_{c}^{*}}+1\right\}}^{\frac{1}{K_{p}-1}}$$ (19)

Therefore, simultaneous Eqs (14), (17), and (19), the displacement of the borehole wall is:

$$u_{0}=2A\left(t\right)r_{0}\left\{\left\{\frac{1}{1+\eta_{1}}+\frac{1}{1+\eta_{2}}\left[{\left(\frac{R_{b}\left(t\right)}{r_{0}}\right)}^{1+\eta_{2}}-1\right]\right\}{\left(\frac{R_{p}\left(t\right)}{R_{b}\left(t\right)}\right)}^{1+\eta_{1}}+\frac{\eta_{1}-1}{2\left(\eta_{1}+1\right)}\right\}$$ (20)

The amount of change in the borehole aperture is:

r0'=r0−2A(t)r0{{11+η1+11+η2[(Rb(t)r0)1+η2−1]}(Rp(t)Rb(t))1+η1+η1−12(η1+1)} (21)

Where *u*_0_ is the displacement of the borehole wall, *r*_0_ is the initial radius of the borehole, *M*_*c*_ is the softening modulus of the coal, $\sigma_{c}^{*}$ is the unidirectional compressive residual strength of the coal, *r*_0_' is the variable quantity over time for borehole aperture.

### 2.2 Negative pressure loss model of drainage

**Fig 2:**
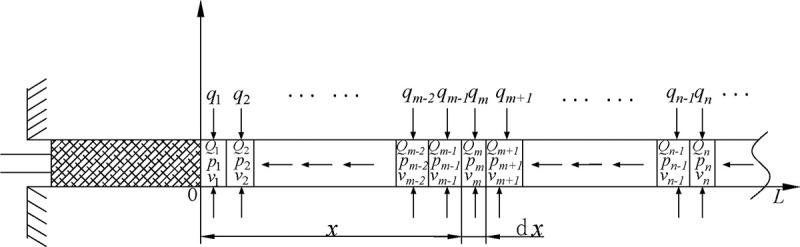
The distribution schematic diagram of micro-element segments in borehole.

Under the action of negative pressure of the drainage, the gas in the coal body around the borehole continuously rushes to the borehole and is discharged through the drainage pipeline. In the process of drainage, the drainage negative pressure will produce pressure loss, and the main factor causing the pressure loss is the friction resistance along the path. In order to facilitate the calculation and analysis, one can assume that the gas in the borehole is driven by pressure gradient; the gas in the coal around the borehole flows into it along the direction of the borehole; the gas flow obeys Darcy's law. The borehole is divided into several micro-element segments along the axial direction of borehole. As shown in Fig 2, the methane gas flows into each micro-element under the negative pressure of drainage.

From the continuity equation of steady flow [23], it can be derived that:

$$Q_{m-1}=Q_{m}+q_{m}$$ (22)

$$v_{m-1}=\frac{Q_{m-1}}{A}=\frac{4Q_{m-1}}{\pi D^{2}}$$ (23)

$$v_{m}=\frac{Q_{m}}{A}=\frac{4Q_{m}}{\pi D^{2}}$$ (24)

$$\bar{v}=\frac{v_{m-1}+v_{m}}{2}=\frac{2\left(2Q_{m}+q_{m}\right)}{\pi D^{2}}$$ (25)

Where *D* is the diameter of the borehole, *A* is the sectional area of a micro-segment, *Q*_*m*_ is the cumulative flow rate of the overflow section, *v*_*m*_ is the gas flow velocity of the mth micro-segment in the borehole, *q*_*m*_ is the flow rate of the gas flowing from the borehole wall into the borehole, $\bar{v}$ is the average gas flow velocity in borehole.

The friction resistance along the path can be expressed as follows:

$$\Delta p_{f}=\frac{f_{m}\rho {\bar{v}}^{2}}{2D}$$ (26)

Where Δ*p*_*f*_ is the loss of frictional resistance, *f*_*m*_ is the friction coefficient of the borehole wall of the mth micro-segment, *ρ* is the gas density of the mth micro-segment.

The pressure loss of the mth micro-segment is:

$$\Delta p_{m}=\Delta p_{f}$$ (27)

$$\frac{dp\left(x\right)}{dx}=\frac{2f_{i}\rho {\left[2Q\left(x\right)+q\left(x\right)\right]}^{2}}{\pi^{2}D^{5}}$$ (28)

Where *p*(*x*) is the pressure *x* meters away from the orifice of the borehole, *f*_*i*_ is the friction resistance factor of the radial flow, *Q*(*x*) is the flow rate from the length *x* of the orifice, *q*(*x*) is the amount of gas flowing from the borehole wall into the borehole.

**Fig 3:**
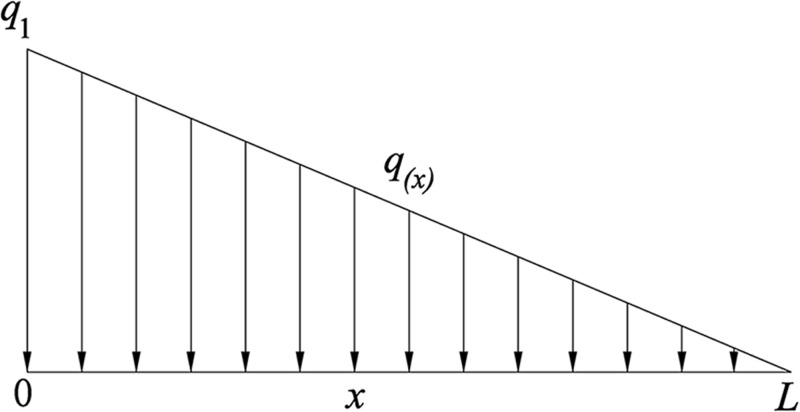
Gas flow model around the borehole.

In order to facilitate the calculation, it is assumed that the attenuation law of the inflow of gas in the coal around the borehole into the borehole follows a linear relationship. It is also considered that the gas inflow of the borehole at the orifice and the end of hole are respectively *q*_1_ and 0, such that the distribution rule of *q*(*x*) in its linear inflow form is obtained, as shown in Fig 3.

$$The\;expression\;of\;q\left(x\right)\;can\;be\;expressed\;as\;q\left(x\right)=\frac{q_{1}}{L}\left(L-x\right)$$ (29)

If the radial flow field radius *R* of the borehole is set to a fixed value, according to the influence radius of the radial flow field in the gas pressure distribution [33], the relationship of flow rate can be obtained by inversely calculating, as shown in Eq (30) below.

$$q\left(t\right)=\left(R-r_{0}\right)\left(p_{0}^{2}-p_{1}^{2}\right)\cdot \sqrt{\frac{\lambda \pi }{2p_{n}p_{0}t}\left(n+\frac{abcp_{0}\left(2+bp_{0}\right)}{{\left(1+bp_{0}\right)}^{2}}\right)}$$ (30)

Where *R* is the radius of influence of the radial flow field, *R* = 3m. *p*_0_ is the original gas pressure of the coal seam, *p*_1_ is the drainage pressure in the coal seam borehole, *p*_*n*_ is the standard atmospheric pressure, *λ* is the permeability coefficient in the coal seam, *a* is the maximum value of the coal adsorption gas, *b* is the gas adsorption constant in the coal seam, *c* is the coal quality parameters, *c* = 1−*A*−*W*, *A* is the ash content of coal, *W* is the coal moisture, *n* is the free gas content of the coal.

According to the theory of fluid dynamics, the gas flow at *x* meters away from the orifice obeys the conservation equation of flow, it can obtain that:

$$\frac{d\left[Q\left(x,t\right)\right]}{dx}=q\left(x,t\right)$$ (31)

According to the above Eqs (28), (29), (30) and (31), the negative pressure loss model of gas drainage borehole can be established.

$$\left\{ \begin{array}{l} \frac{dp\left(x\right)}{dx}=\frac{2f_{i}\rho {\left[2Q\left(x\right)+q\left(x\right)\right]}^{2}}{\pi^{2}D^{5}}\\ q\left(x\right)=\frac{q_{1}}{L}\left(L-x\right)\\ q\left(t\right)=\left(R-r_{0}\right)\left(p_{0}^{2}-p_{1}^{2}\right)\cdot \sqrt{\frac{\lambda \pi }{2p_{n}p_{0}t}\left(n+\frac{abcp_{0}\left(2+bp_{0}\right)}{{\left(1+bp_{0}\right)}^{2}}\right)}\\ \frac{d\left[Q\left(x,t\right)\right]}{dx}=q\left(x,t\right) \end{array}\right.$$ (32)

By solving the pressure loss model, the negative pressure distribution formula *p*(*x*,*t*) in borehole is obtained as below, which is in the form of the gas linear profile inflow.

$$p\left(x,t\right)=-\frac{2f_{i}\rho q^{2}\left(t\right)}{\pi^{2}d^{5}L^{2}}\left\{\frac{1}{5}\left[L^{5}+{\left(x-L\right)}^{5}\right]+\frac{1}{2}\left[L^{4}-{\left(x-L\right)}^{4}\right]+\frac{1}{3}\left[L^{3}+{\left(x-L\right)}^{3}\right]\right\}+p_{1}$$ (33)

At the same time, the borehole creep Eq (21) is substituted into Eq (33) to obtain the formula of instability-negative pressure loss of gas drainage borehole.

$$p{\left(x,t\right)}^{\prime }=-\frac{f_{i}\rho {q^{\prime }}^{2}\left(x,t\right)}{16\pi^{2}{r^{\prime }}_{0}^{5}L^{2}}\left\{\frac{1}{5}\left[L^{5}+{\left(x-L\right)}^{5}\right]+\frac{1}{2}\left[L^{4}-{\left(x-L\right)}^{4}\right]+\frac{1}{3}\left[L^{3}+{\left(x-L\right)}^{3}\right]\right\}+p_{1}$$ (34)

## 3. Solving process of the model

### 3.1 Analysis on the stress distribution around roadway

**Fig 4:**
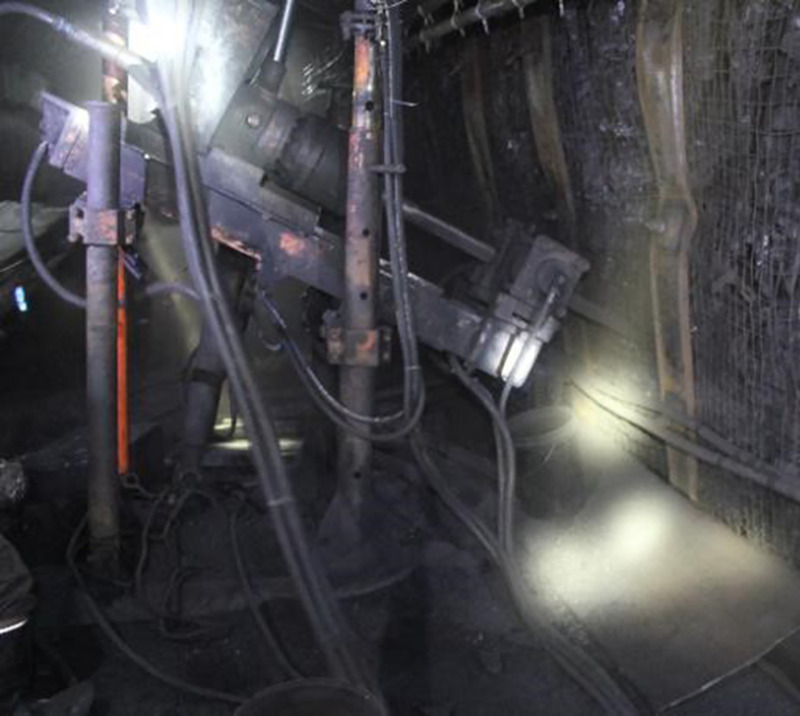
Physical picture of drill cuttings field test in the coal mine.

The cuttings of six boreholes are measured in the middle roadway of 24130 coal face in Pingmei No.10 coal mine and the mean value is obtained. The field test is shown in Fig 4. According to the formula for estimating surrounding rock pressure established by Zhao et al. [34], the stress around roadway *p*_2_ is obtained through repeated trials using the approximation method. The amount of drilling cuttings *G* is expressed as follows.

$$G=\gamma \pi lr_{0}{}^{2}\left\{\frac{1+\nu_{0}}{E_{0}}\times {\left\{\frac{\sigma_{c}{\left[1+(q-1)-\frac{\sigma_{0}}{\sigma_{c}}\right]}^{m}{\left(\frac{1+\nu_{0}}{E_{0}}\right)}^{m}{\left(\frac{E}{1+\nu }\right)}^{m}\frac{1}{2m+q-1}+\frac{2\sigma_{0}-\sigma_{c}}{1+q}}{\sigma_{c}{\left[1+(q-1)-\frac{\sigma_{0}}{\sigma_{c}}\right]}^{m}{\left(\frac{1+\nu_{0}}{E_{0}}\right)}^{m}{\left(\frac{E}{1+\nu }\right)}^{m}\frac{1}{2m+q-1}}\right\}}^{\frac{2}{2m+q-1}}\times \left[\sigma_{c}+\frac{q-1}{q+1}(2\sigma_{0}-\sigma_{c})\right]+1\right\}$$ (35)

Where *γ* is the bulk density of the surrounding rock, *l* is the depth of the hole, *r*_0_ is the initial radius of the borehole. The remaining parameters are well introduced in the reference 34.

**Fig 5:**
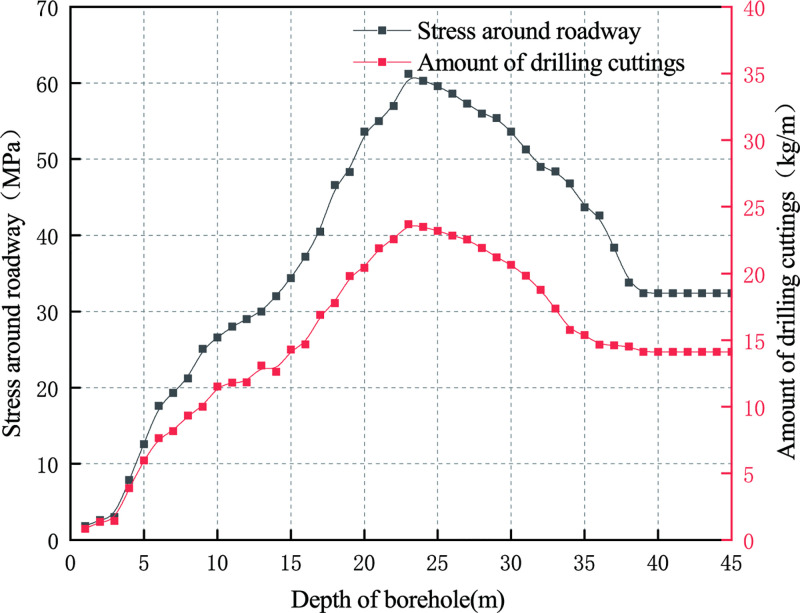
Evolution of the amount of drilling cuttings and the pressure of surrounding rock with the depth of borehole.

According to the measured amount of drilling cuttings and calculated stress around roadway, the evolution of the amount of drilling cuttings and the stress around roadway with the depth of borehole can be derived, as shown in Fig 5. It can be seen from the figure that both the amount of drilling cuttings and the pressure of surrounding rock first increase to the maximum, then decrease, and then tend to stabilize, and the relationship between the two shows a positive correlation. According to the dynamic phenomenon generated during the drill cuttings at the site and Fig 5, one can observes that the distribution trend of the surrounding rock pressure in the range of 0m~39m firstly rise, then decline, and before it gradually stabilizes. The stress rise, which occurs from 0 to 13m, is relatively stable, which corresponds to the crushing zone of the roadway. The stress concentration area of the roadway is from 14m to 39m, the peak value of the pressure of surrounding rock at the roadway occurs at 23m, which reaches the value of 61.2MPa. After 40m, the pressure of surrounding rock becomes relatively stable, which is the original rock stress zone of the roadway. The stress concentration factor is 1.9.

### 3.2 Analysis on the creep variation of the coal around borehole

Taking the 24130 coal face in Pingmei No.10 coal mine as an example, the buried depth of the coal seam is about 1200m, the coal thickness is 2.2m ~3.5m, the sealing depth of borehole is 15 m, the sealing section length of borehole is 8 m, the detailed parameters are shown in Table 1. Use the mathematical software MATLAB 2018a and Origin 2018 to solve the model.

**Table 1:** Table of calculation parameters.

The parameter name	Parameter	Values and units
Compressive strength of the coal	*σ*_*c*_	4.1MPa
Instantaneous shear modulus	*G*_0_	1000MPa
Long-term shear modulus	*G*_∞_	500MPa
The radius of the borehole	*r*_0_	0.05m
Internal friction angle of coal	*φ*	25°
Expansion coefficient in the plastic softening zone	*η*_1_	3
Expansion coefficient in the crushing zone	*η*_2_	1.3
The softening modulus of the coal	*M*_*c*_	2320MPa
Unidirectional compressive residual strength	$\sigma_{c}^{*}$	0.21MPa
Bulk density of the surrounding rock	*γ*	27kN/m^3^
Radius of influence of the radial flow field	*R*	3m
Original gas pressure of the coal seam	*p*_0_	1.5MPa
Drainage pressure in the coal seam borehole	*p*_1_	20kPa
Standard atmospheric pressure	*p*_*n*_	0.1MPa
Permeability coefficient in the coal seam	*λ*	0.0106m^2^/(Pa^2^·s)
Maximum value of the coal adsorption gas	*a*	22.67cm^3^/g
Gas adsorption constant in the coal seam	*b*	0.6 MPa^-1^
Ash content of coal	*A*	17.88%
Coal moisture	*W*	0.78
Free gas content of the coal	*n*	1m^3^/t
Gas density	*ρ*	0.7kg/m^3^
Friction coefficient of the borehole wall	*f*_*i*_	0.17

**Fig 6:**
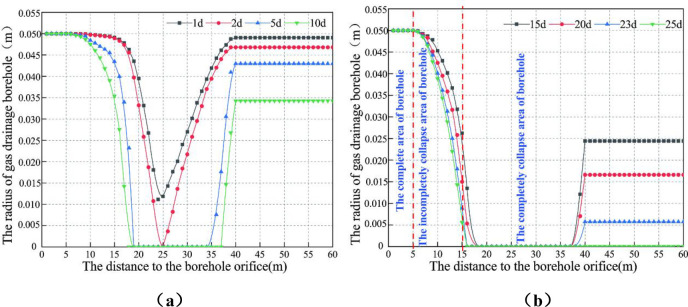
The relationship of the gas drainage borehole aperture as a function of time.

The mathematical software was used to solve the Formula (21), and the variation law of borehole aperture with time was plotted, as shown in Fig 6 (S1 Table in S1 File).

Within the first two days after the completion of the borehole construction, the collapsed area inside the borehole is mainly distributed between 14 and 36 m. The borehole collapse is the most serious at the stress peak of 23m, as shown in Fig 6(A).

The total length of the gas drainage borehole is 80m, which is respectively distributed in the crushing zone, the stress concentration zone and the original rock stress zone of the roadway. Through analysis, it can be seen from Fig 6 that:

Over time, the borehole collapse area would expand to the crushing zone and the original rock stress zone. On the 5th day after the completion of the borehole construction, the borehole in the stress concentration zone completely collapses from 18 to 34 m. On the 10th day, the complete collapse area of the borehole ranges from 17 to 36 m. The complete collapse of the borehole on the 25th day ranges from 15 m to the final hole of the borehole.

After 25 days, the coal around the borehole eventually forms three zones along the length of the borehole, as shown in Fig 6(B). From the orifice of the borehole to the inner part of the borehole, 0 ~ 5m is the complete area of borehole, 5 ~ 15m is the incompletely collapse area of borehole, and from 15 to the final hole is the complete collapse area of borehole.

In summary, the gas drainage borehole of the deep coal seams is in a complex mechanical environment. The borehole starts to show different degrees of collapse on the first day after completion of the construction. The collapsed area of the borehole started to extend from the peak of stress concentration to both ends. At 25 days, almost the entire borehole collapse except that the borehole orifice is not collapsed within 5m.

**Fig 7:**
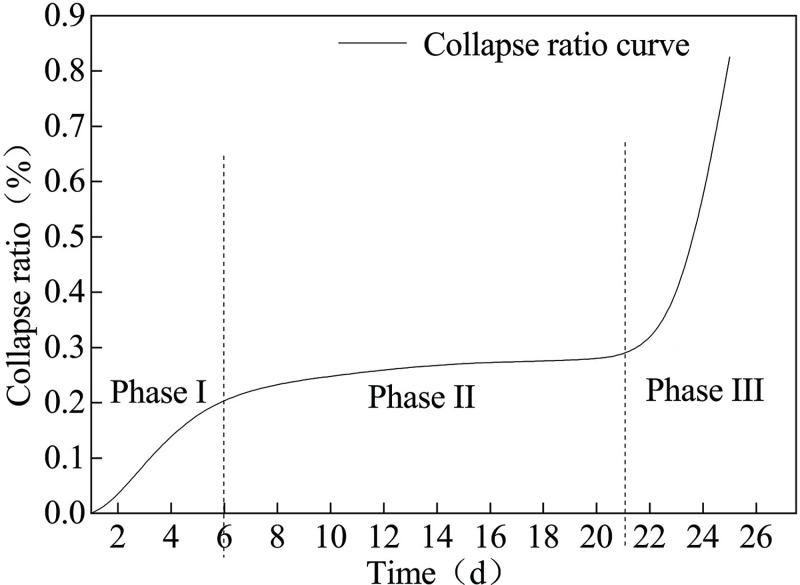
Three stages of borehole collapse ratio.

The collapse ratio of the borehole can be determined according to the ratio of the collapsed portion of the borehole to the total bore length of the borehole. The associated plot is shown in Fig 7 (S2 Table in S1 File).

According to Fig 7, one can see that the collapse ratio of the borehole is rising rapidly in the first six days. From the 7^th^ to the 21^st^ days, the rise generally proceeds, but the increasing rate of the collapse ratio lowers significantly. The collapse ratio thereafter rapidly increases after 21 days. Therefore, the evolution of the collapse ratio is divided into three stages. In phase I, the crushing zone, the plastic zone and the elastic zone of the borehole are formed due to the redistribution of the stress around the borehole after its formation. The stress in the elastoplastic zone of the borehole is significantly lower than the stress in the stress concentration zone of the roadway. The borehole in the stress concentration zone is unstable and collapses rapidly. During phase II, the borehole in the stress concentration zone will be compacted after the collapse, and the borehole in the original rock stress zone has creeped but not collapsed. During Phase III, the reason for the rapid rise of the collapse ratio curve is that the borehole in the stress zone of the original rock is affected by the stress, which induces creep deformation and eventually leads to more instability and collapses.

**Fig 8:**
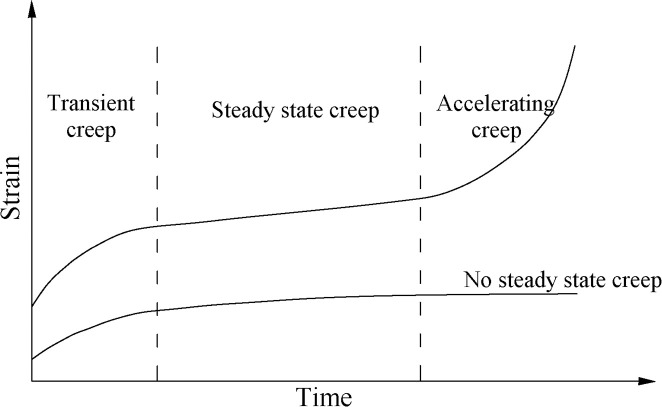
Three stages of a typical creep curve under stress.

It should be noted that the three stages of the collapse ratio change with time are approximately similar to the three stages of the creep [15, 35], as shown in Fig 8. The higher the stress level, the greater the creep deformation. In the process of transient creep, the stress is greater than the instantaneous strength limit of coal and rock, and the coal is damaged instantaneously. When the stress on coal is greater than long-term strength limit and less than instantaneous strength limit, stable creep occurs. When the stress on coal is less than the long-term strength limit, the coal will creep in unstable state. In the process of deformation, long-term strength plays an important role. When the stress level is higher than the long-term strength, the long or short time will eventually lead to the rock rupture, the three stages of the creep process are all included. Otherwise, when the stress level is lower than the long-term strength, only the former two stages are all included. Therefore, the formation process of collapse ratio curve is similar to that of typical creep curve of rock, which is the displacement change of stress intensity on coal and rock with time.

### 3.3 Variation law of negative pressure loss in borehole

**Fig 9:**
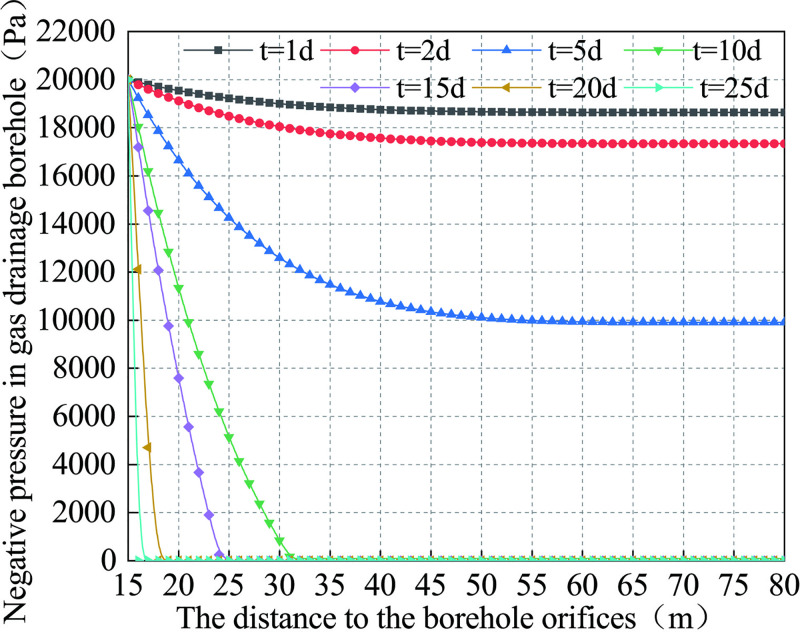
Curve of the negative pressure change of gas drainage borehole.

According to Eq (34), the relationship between the drainage negative pressure distribution in the borehole can be plotted against time, as shown in Fig 9 (S3 Table in S1 File).

Referring to Figs 6 and 9, one may see that on the first day of the gas drainage, the incomplete collapse occurs in the range of 15~35 m of the drainage borehole, and the pressure loss of the drainage borehole is 1.36 kPa. On the fifth day, the borehole collapses at 18~34 m; the pressure drops rapidly at 15~35m. The pressure loss is 8.5kPa, and the pressure loss decreases slowly from 35m to the final hole. On the 10th day, the borehole collapses at 17~36m, and the middle part of the collapse area is further compacted at 23m. The pressure decreases rapidly from 15 to 31m until it nullifies. By the 25th day, the negative pressure in the borehole is nearly zero, and there is almost no negative pressure in the borehole.

The analysis suggests that the main cause of the negative pressure loss of the borehole is the collapse deformation of the borehole. The collapse inside the borehole leads to the negative pressure loss in the borehole, which subsequently hinder significantly the gas drainage transport channel and renders difficult the extraction of gas in the borehole. Therefore, in order to improve the concentration of gas drainage in borehole, it is necessary to control the creep deformation of the borehole and reduce the negative pressure loss in the borehole.

## 4. Prevention technology

**Fig 10:**
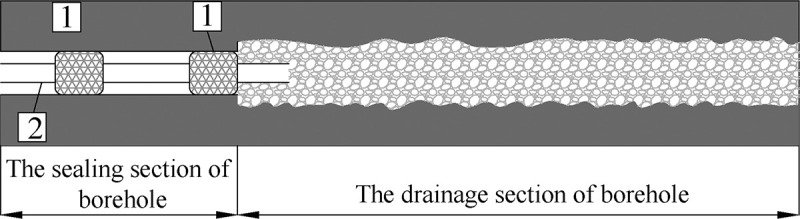
Schematic diagram of collapse in drainage section of borehole. 1—The sealing device of “two sealing and one grouting”, 2—Drainage pipe.

**Fig 11:**
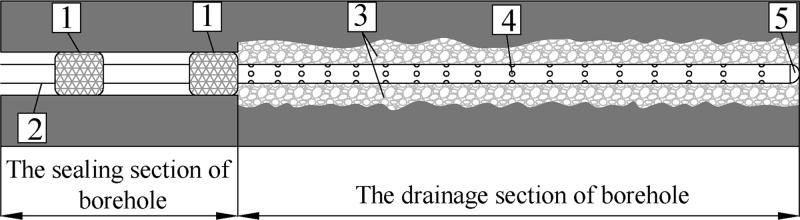
Structure principle of the FDSMP sealing device. 1—The sealing device of “two sealing and one grouting”, 2—Drainage straight pipe, 3—The collapsed coal around borehole, 4—Drainage screen mesh pipe, 5—Plastic stopper.

The traditional drainage technology cannot provide mechanical support for the boreholes. Creep collapse occurs when the borehole cannot support complex stress changes, and the extraction pipe is easily blocked by crushed coal, as shown in Fig 10. For this reason, this study proposes a prevention technology, called of “Full-hole deep screen mesh pipe” (Hereinafter referred to as FDSMP) to control the creep deformation and the negative pressure loss of the borehole, as shown in Fig 11.

### 4.1 The structure design

**Fig 12:**
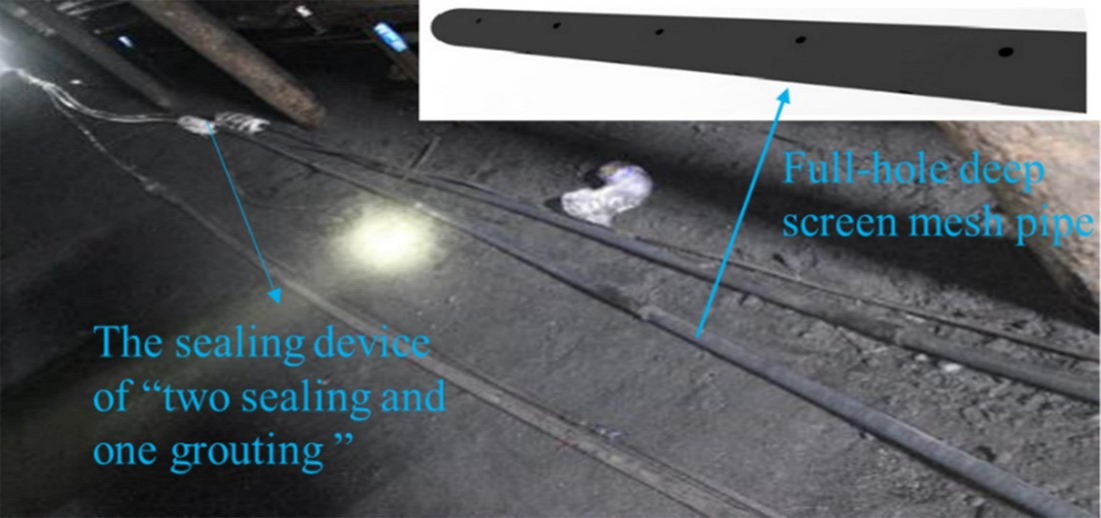
The physical picture of FDSMP.

The “straight pipe” in “FDSMP” is a drainage pipe that is positioned in the sealing borehole section. The “screen mesh pipe” is placed in the entire section of the drainage section of the borehole, which has the function to reduce the loss of negative pressure and control the deformation of coal around the borehole. The key technology is to put a section of screen mesh pipe into the drainage section of the borehole. The layout of the screen mesh pipe is shown in Figs 11 and 12. The diameter of the screen mesh pipe is the same as that of the drainage pipe, the drainage pipe and the screen mesh pipe forms one single continuous structure. The diameter of the sieve hole on the screen mesh pipe is 6mm, and the sieve hole on the screen mesh pipe evenly arranges 20 per meter.

### 4.2 Analysis of the law of negative pressure loss in prevention technology

**Fig 13:**
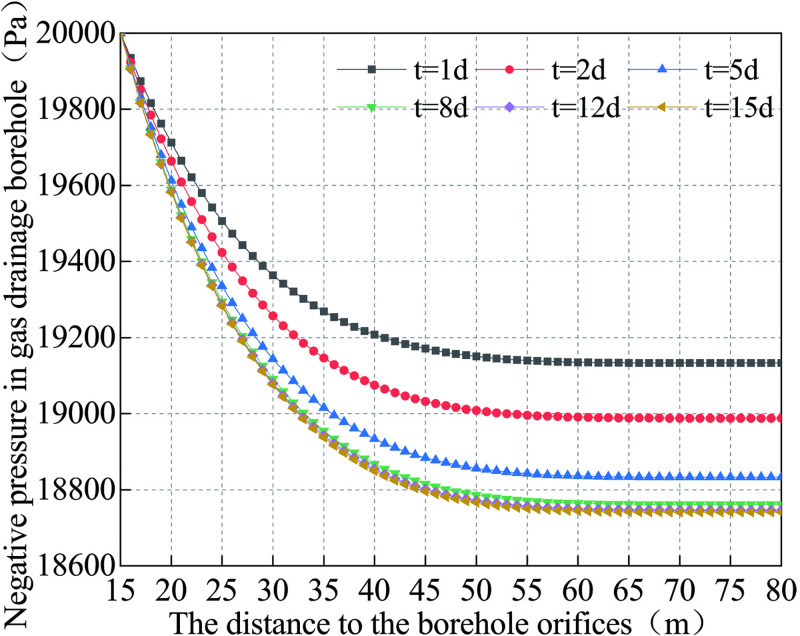
Curve of the negative pressure loss in prevention technology.

**Fig 14:**
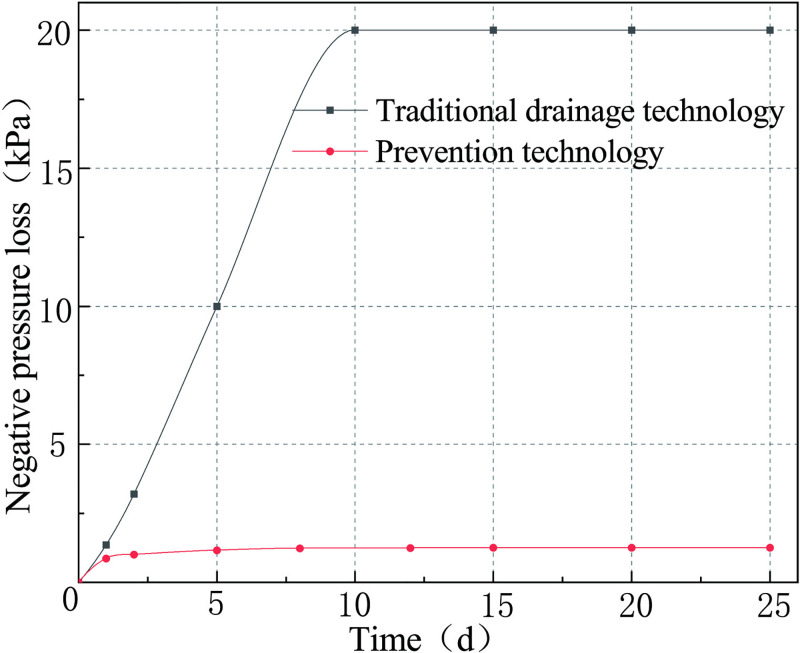
Variation curve of negative pressure loss in gas drainage borehole.

As the coal surrounding the borehole is affected by the pressure of the coal seam and the creep properties of the coal, the borehole diameter of the borehole will decrease with time until the coal around the borehole is squeezed onto the screen mesh pipe. This will enable the screen mesh pipe and the surrounding coal to reach a new equilibrium of force. According to the formula (34), the evolution of negative pressure loss in full-hole deep screen mesh pipe as a function of the borehole length was plotted, as shown in Fig 13 (S4 Table in S1 File). By analyzing the negative pressure loss curves of borehole of the two drainage technologies in Figs 9 and 13, the curve of negative pressure loss with time of the two drainage technologies can be derived, as shown in Fig 14 (S5 Table in S1 File).

Fig 13 shows that with the increase of the distance from the orifice of the borehole, the negative pressure in the FDSMP decreases continuously, but the rate of decline continuously decreases too. On the first day of the gas drainage, the pressure loss is 0.87 kPa. The pressure loss is then 1.26 kPa when it reaches stability on the 15th day. Simultaneously, one can also see in Fig 14 that the negative pressure loss in the borehole under the traditional drainage technology increases linearly in the first 10 days, while the negative pressure loss of the prevention technology increases very slowly. On the first day, the negative pressure loss of traditional drainage technology is 1.56 times that of the prevention technology. By the 10th day, the negative pressure loss of traditional drainage technology is 8 times that of the prevention technology. After 15 days, the pressure loss ratio of the two drainage technologies reaches 15.8. The comparison shows that the prevention technology can significantly reduce the negative pressure loss in the borehole, increase the drainage concentration of gas in the borehole, and effectively shorten the drainage period.

### 4.3 Effect analysis

**Fig 15:**
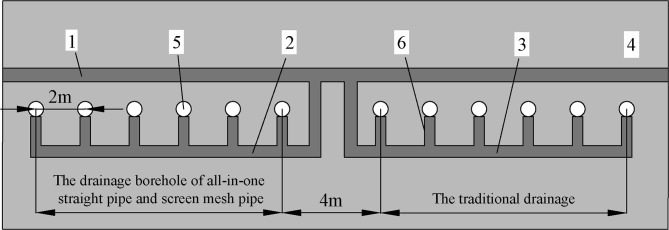
The schematic diagram of the drainage borehole layout. 1—The main gas drainage pipe, 2—Drainage tandem pipe of new technology, 3—Drainage tandem pipe of traditional technology, 4—Coal seam, 5—Borehole, 6—The connecting pipe.

The sealing depth of the two drainage technologies is 15m, the length of the sealing section is 8m, the diameter of the drainage pipe is 50mm, the depth of the borehole is 80m, and among them, a 60m length screen mesh pipe is arranged in the drainage section of the prevention technology. Six boreholes are arranged for each of the two drainage technologies, and the two sets of boreholes are connected to the main gas drainage pipe by a connecting pipe the same diameter as extraction pipe, as shown in Fig 15 below. An observation hole is set on the connecting pipe of each borehole to detect the concentration of gas in the borehole. An orifice flowmeter is connected to each group of boreholes to measure the gas flow.

**Fig 16:**
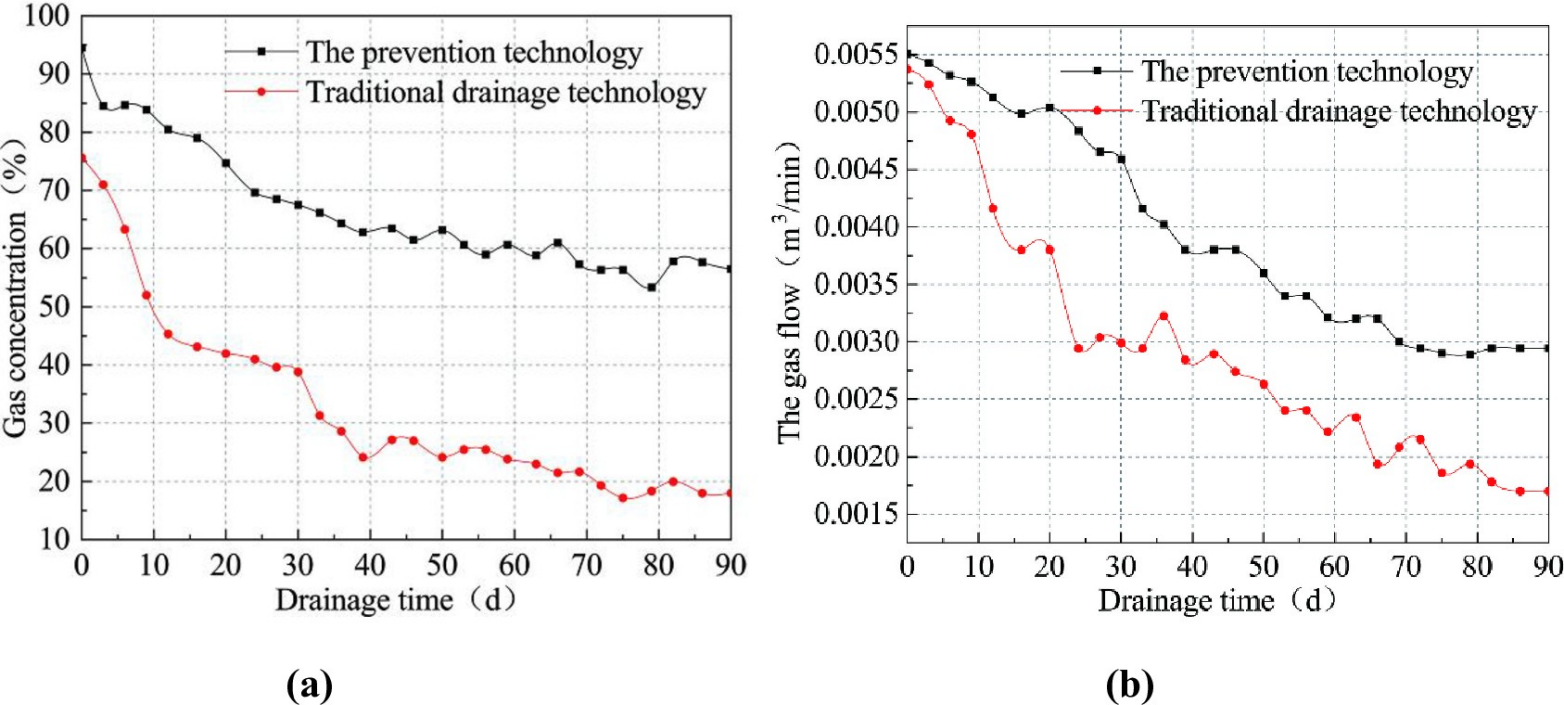
Evolution curve of the average gas drainage concentration (left) and the gas flow (right) with drainage time.

After sealing the borehole, the gas concentration and flow of the test borehole are monitored. The time of drainage is 90 days. The data of gas the concentration and the gas flow are collected separately for each set of boreholes. The results of the comparison within 90 days are shown in Fig 16 (S6 Table in S1 File).

The comparison of gas drainage concentration between the two drainage technologies in Fig 16(A) shows that, the trend of the curve for the prevention technology is relatively stable in the 20 days to 90 days, during which the decreasing rate is not very high. While the traditional drainage technology is relatively unstable, with a high decreasing rate.

Analysis of the drainage concentration data can be found that, the average gas concentration of the borehole in the prevention technology is 78.7% within 30 days, while the average gas concentration of the borehole in the traditional drainage technology is 51.2%. The average gas concentration of prevention technology is increased by 53.7% relative to the traditional. After 90 days of drainage, the average gas concentration of the borehole measured by the prevention technology is 66.6%, and the average gas concentration of the borehole in the traditional drainage technology is 33.1%. The average gas concentration of the prevention technology is increased by 101% compared with the traditional. Furthermore, it can be seen from Fig 16(B) that the gas drainage flow of the prevention technology is higher than that of the traditional drainage technology. The highest ratio of gas flow in two drainage technologies is 65.5%, and the average drainage flow rate is increased by 33.3%.

The gas concentration of the prevention technology and the traditional drainage technology shows a decreasing trend with time. However, the gas concentration and the flow of the borehole in the traditional drainage technology decrease rapidly within 10 days. This indicates that the drainage section in the borehole with the traditional drainage technology has collapsed within 10 days, which caused great influence on the gas drainage in borehole. The concentration of gas drainage in the borehole decreases relatively gently with the prevention technology, it indicates that the collapse of the drainage section in the borehole has little influence with the prevention technology. In summary, through the comparative tests, one can see that the prevention technology can effectively reduce the negative pressure loss of drainage in the boreholes, increase the gas drainage concentration in boreholes and shorten the gas drainage cycle in coal seams. It can be seen that the test results verify the correctness of the above theoretical analysis. This new technique presents obvious advantages compared with the traditional.

## 5. Conclusion

In order to better understand the collapse deformation and the negative pressure loss of the gas drainage boreholes in deep coal seams, creep model of coal around borehole and negative pressure loss model are established. The variation law of borehole diameter and negative pressure loss is deeply analysed, and verified by engineering tests. Based on the results of this study, the following conclusions can be drawn:

The creep model of coal around borehole is established based on the plastic softening characteristics of coal. Three stages of borehole creep in deep coal seams are proposed, namely the three stages theory of collapse ratio. In phase I, the borehole in the stress concentration zone of the roadway firstly collapses rapidly. With the passage of time, and the collapse area of borehole extends to both ends of the borehole over time. In phase II, the borehole in the stress concentration zone collapses completely and has been compacted, and the borehole in the original rock stress zone only experiences creep. In phase III, the borehole in the original rock stress zone is instability and collapses. After three stages, the inside of borehole eventually collapses completely and forms three areas, namely the complete area, the incomplete collapse area, and the completely collapsed area. This can provide a technical reference for optimizing the sealing process of drainage borehole.

According to the coal collapse creep model around the borehole, the gas radial flow field model and the borehole gas flow model, the model of instability-negative pressure loss model of gas drainage borehole is established. The negative pressure loss in borehole under traditional and prevention technology is analyzed theoretically, and the main factor for the negative pressure loss of drainage borehole is found to be the collapse of the borehole. The mathematical model provides a theoretical basis for the study of negative pressure loss in borehole.

Compared with the traditional drainage technology, the negative pressure in the gas drainage borehole can be transferred to the entire drainage section of the borehole under the prevention technology. And it can provide mechanical support for the drainage section of borehole, greatly reduce the collapse of the borehole, effectively reduce the negative pressure loss of drainage borehole. The prevention technology is more conducive to the gas drainage, it can increase the gas drainage concentration in the borehole, significantly improve the coal seam gas drainage effect and shorten the drainage cycle. The research results can provide powerful theoretical guidance for gas drainage in deep coal seams.

## Supporting information

S1 File (DOCX)
