# Supplementary material for: Instability-negative pressure loss model of gas drainage borehole and prevention technique: A case study
Source: PLoS One. 2020 Nov 23;15(11):e0242719. doi: 10.1371/journal.pone.0242719 (PMC7682895; doi:10.1371/journal.pone.0242719)
Supplement: S1 File — (DOCX) [file pone.0242719.s001.docx]

**S1 Table. The calculated data of gas drainage radius in axial direction of borehole**

| **Distance**  **(m)** | **The radius of gas drainage borehole (m)** | | | | | | | |
| --- | --- | --- | --- | --- | --- | --- | --- | --- |
|  | **1d** | **2d** | **5d** | **10d** | **15d** | **20d** | **23d** | **25d** |
| 1 | 0.050 | 0.050 | 0.050 | 0.050 | 0.050 | 0.050 | 0.050 | 0.050 |
| 2 | 0.050 | 0.050 | 0.050 | 0.050 | 0.050 | 0.050 | 0.050 | 0.050 |
| 3 | 0.050 | 0.050 | 0.050 | 0.050 | 0.050 | 0.050 | 0.050 | 0.050 |
| 4 | 0.050 | 0.050 | 0.050 | 0.050 | 0.050 | 0.050 | 0.050 | 0.050 |
| 5 | 0.050 | 0.050 | 0.050 | 0.050 | 0.050 | 0.050 | 0.050 | 0.050 |
| 6 | 0.050 | 0.050 | 0.050 | 0.050 | 0.050 | 0.050 | 0.050 | 0.050 |
| 7 | 0.050 | 0.050 | 0.050 | 0.050 | 0.049 | 0.049 | 0.049 | 0.049 |
| 8 | 0.050 | 0.050 | 0.050 | 0.049 | 0.049 | 0.048 | 0.047 | 0.047 |
| 9 | 0.050 | 0.050 | 0.049 | 0.049 | 0.048 | 0.045 | 0.044 | 0.043 |
| 10 | 0.050 | 0.050 | 0.048 | 0.048 | 0.045 | 0.043 | 0.040 | 0.039 |
| 11 | 0.050 | 0.050 | 0.048 | 0.046 | 0.043 | 0.040 | 0.036 | 0.034 |
| 12 | 0.050 | 0.050 | 0.047 | 0.044 | 0.040 | 0.036 | 0.031 | 0.029 |
| 13 | 0.050 | 0.049 | 0.046 | 0.042 | 0.037 | 0.032 | 0.025 | 0.022 |
| 14 | 0.049 | 0.049 | 0.046 | 0.039 | 0.032 | 0.026 | 0.018 | 0.014 |
| 15 | 0.049 | 0.049 | 0.043 | 0.035 | 0.026 | 0.015 | 0.009 | 0.006 |
| 16 | 0.049 | 0.048 | 0.040 | 0.028 | 0.013 | 0.005 | 0.000 | 0.000 |
| 17 | 0.048 | 0.047 | 0.034 | 0.014 | 0.000 | 0.000 | 0.000 | 0.000 |
| 18 | 0.046 | 0.046 | 0.023 | 0.000 | 0.000 | 0.000 | 0.000 | 0.000 |
| 19 | 0.044 | 0.040 | 0.000 | 0.000 | 0.000 | 0.000 | 0.000 | 0.000 |
| 20 | 0.039 | 0.033 | 0.000 | 0.000 | 0.000 | 0.000 | 0.000 | 0.000 |
| 21 | 0.033 | 0.026 | 0.000 | 0.000 | 0.000 | 0.000 | 0.000 | 0.000 |
| 22 | 0.027 | 0.019 | 0.000 | 0.000 | 0.000 | 0.000 | 0.000 | 0.000 |
| 23 | 0.018 | 0.011 | 0.000 | 0.000 | 0.000 | 0.000 | 0.000 | 0.000 |
| 24 | 0.011 | 0.000 | 0.000 | 0.000 | 0.000 | 0.000 | 0.000 | 0.000 |
| 25 | 0.012 | 0.000 | 0.000 | 0.000 | 0.000 | 0.000 | 0.000 | 0.000 |
| 26 | 0.014 | 0.004 | 0.000 | 0.000 | 0.000 | 0.000 | 0.000 | 0.000 |
| 27 | 0.018 | 0.008 | 0.000 | 0.000 | 0.000 | 0.000 | 0.000 | 0.000 |
| 28 | 0.020 | 0.014 | 0.000 | 0.000 | 0.000 | 0.000 | 0.000 | 0.000 |
| 29 | 0.024 | 0.018 | 0.000 | 0.000 | 0.000 | 0.000 | 0.000 | 0.000 |
| 30 | 0.027 | 0.022 | 0.000 | 0.000 | 0.000 | 0.000 | 0.000 | 0.000 |
| 31 | 0.030 | 0.026 | 0.000 | 0.000 | 0.000 | 0.000 | 0.000 | 0.000 |
| 32 | 0.034 | 0.030 | 0.000 | 0.000 | 0.000 | 0.000 | 0.000 | 0.000 |
| 33 | 0.037 | 0.033 | 0.000 | 0.000 | 0.000 | 0.000 | 0.000 | 0.000 |
| 34 | 0.039 | 0.037 | 0.000 | 0.000 | 0.000 | 0.000 | 0.000 | 0.000 |
| 35 | 0.042 | 0.039 | 0.000 | 0.000 | 0.000 | 0.000 | 0.000 | 0.000 |
| 36 | 0.044 | 0.042 | 0.008 | 0.000 | 0.000 | 0.000 | 0.000 | 0.000 |
| 37 | 0.046 | 0.044 | 0.018 | 0.000 | 0.000 | 0.000 | 0.000 | 0.000 |
| 38 | 0.047 | 0.046 | 0.031 | 0.015 | 0.000 | 0.000 | 0.000 | 0.000 |
| 39 | 0.049 | 0.047 | 0.041 | 0.027 | 0.012 | 0.007 | 0.000 | 0.000 |
| 40 | 0.049 | 0.047 | 0.043 | 0.034 | 0.024 | 0.017 | 0.006 | 0.000 |
| 41 | 0.049 | 0.047 | 0.043 | 0.034 | 0.024 | 0.017 | 0.006 | 0.000 |
| 42 | 0.049 | 0.047 | 0.043 | 0.034 | 0.024 | 0.017 | 0.006 | 0.000 |
| 43 | 0.049 | 0.047 | 0.043 | 0.034 | 0.024 | 0.017 | 0.006 | 0.000 |
| 44 | 0.049 | 0.047 | 0.043 | 0.034 | 0.024 | 0.017 | 0.006 | 0.000 |
| 45 | 0.049 | 0.047 | 0.043 | 0.034 | 0.024 | 0.017 | 0.006 | 0.000 |
| 46 | 0.049 | 0.047 | 0.043 | 0.034 | 0.024 | 0.017 | 0.006 | 0.000 |
| 47 | 0.049 | 0.047 | 0.043 | 0.034 | 0.024 | 0.017 | 0.006 | 0.000 |
| 48 | 0.049 | 0.047 | 0.043 | 0.034 | 0.024 | 0.017 | 0.006 | 0.000 |
| 49 | 0.049 | 0.047 | 0.043 | 0.034 | 0.024 | 0.017 | 0.006 | 0.000 |
| 50 | 0.049 | 0.047 | 0.043 | 0.034 | 0.024 | 0.017 | 0.006 | 0.000 |
| 51 | 0.049 | 0.047 | 0.043 | 0.034 | 0.024 | 0.017 | 0.006 | 0.000 |
| 52 | 0.049 | 0.047 | 0.043 | 0.034 | 0.024 | 0.017 | 0.006 | 0.000 |
| 53 | 0.049 | 0.047 | 0.043 | 0.034 | 0.024 | 0.017 | 0.006 | 0.000 |
| 54 | 0.049 | 0.047 | 0.043 | 0.034 | 0.024 | 0.017 | 0.006 | 0.000 |
| 55 | 0.049 | 0.047 | 0.043 | 0.034 | 0.024 | 0.017 | 0.006 | 0.000 |
| 56 | 0.049 | 0.047 | 0.043 | 0.034 | 0.024 | 0.017 | 0.006 | 0.000 |
| 57 | 0.049 | 0.047 | 0.043 | 0.034 | 0.024 | 0.017 | 0.006 | 0.000 |
| 58 | 0.049 | 0.047 | 0.043 | 0.034 | 0.024 | 0.017 | 0.006 | 0.000 |
| 59 | 0.049 | 0.047 | 0.043 | 0.034 | 0.024 | 0.017 | 0.006 | 0.000 |
| 60 | 0.049 | 0.047 | 0.043 | 0.034 | 0.024 | 0.017 | 0.006 | 0.000 |

**S2 Table. The data on collapse ratio over time**

| **Time**  **d** | **Collapse length**  **m** | **Collapse ratio**  **%** | **Time**  **d** | **Collapse length**  **m** | **Collapse ratio**  **%** |
| --- | --- | --- | --- | --- | --- |
| 1 | 0 | 0 | 15 | 22 | 0.275 |
| 2 | 2 | 0.025 | 20 | 22 | 0.275 |
| 5 | 17 | 0.2125 | 23 | 24 | 0.3 |
| 10 | 20 | 0.25 | 25 | 66 | 0.825 |

**S3 Table.** **Negative pressure value in axial direction of gas drainage borehole** **under traditional drainage technology**

| **Distance**  **(m)** | **Negative pressure in gas drainage borehole（Pa）** | | | | | | |
| --- | --- | --- | --- | --- | --- | --- | --- |
|  | **t=1d** | **t=2d** | **t=5d** | **t=10d** | **t=15d** | **t=20d** | **t=25d** |
| 15 | 20000 | 20000 | 20000 | 20000 | 20000 | 20000 | 20000 |
| 16 | 19897.74637 | 19800.48058 | 19241.2907 | 18038.87586 | 17191.07799 | 12112.05685 | 0 |
| 17 | 19801.73537 | 19613.1419 | 18528.90092 | 16197.47918 | 14553.64186 | 4705.676 | 0 |
| 18 | 19711.67671 | 19437.41754 | 17860.67671 | 14470.24239 | 12079.71716 | 0 | 0 |
| 19 | 19627.28922 | 19272.7589 | 17234.53196 | 12851.77324 | 9761.58057 | 0 | 0 |
| 20 | 19548.30075 | 19118.63495 | 16648.44727 | 11336.85202 | 7591.75589 | 0 | 0 |
| 21 | 19474.448 | 18974.53191 | 16100.46895 | 9920.42874 | 5563.01001 | 0 | 0 |
| 22 | 19405.47636 | 18839.95299 | 15588.70786 | 8597.62036 | 3668.34893 | 0 | 0 |
| 23 | 19341.13978 | 18714.4181 | 15111.33836 | 7363.70791 | 1901.01368 | 0 | 0 |
| 24 | 19281.20062 | 18597.46356 | 14666.59722 | 6214.13378 | 254.47636 | 0 | 0 |
| 25 | 19225.42952 | 18488.64182 | 14252.78253 | 5144.49884 | 0 | 0 | 0 |
| 26 | 19173.60522 | 18387.52116 | 13868.25261 | 4150.55965 | 0 | 0 | 0 |
| 27 | 19125.51444 | 18293.68542 | 13511.42494 | 3228.2257 | 0 | 0 | 0 |
| 28 | 19080.95172 | 18206.73371 | 13180.77507 | 2373.55654 | 0 | 0 | 0 |
| 29 | 19039.71929 | 18126.28012 | 12874.83552 | 1582.75901 | 0 | 0 | 0 |
| 30 | 19001.62691 | 18051.95346 | 12592.19471 | 852.18444 | 0 | 0 | 0 |
| 31 | 18966.49171 | 17983.39692 | 12331.49586 | 178.32583 | 0 | 0 | 0 |
| 32 | 18934.13808 | 17920.26785 | 12091.43594 | 0 | 0 | 0 | 0 |
| 33 | 18904.39751 | 17862.23741 | 11870.76453 | 0 | 0 | 0 | 0 |
| 34 | 18877.10841 | 17808.99035 | 11668.28279 | 0 | 0 | 0 | 0 |
| 35 | 18852.11601 | 17760.22467 | 11482.84232 | 0 | 0 | 0 | 0 |
| 36 | 18829.27221 | 17715.65136 | 11313.34412 | 0 | 0 | 0 | 0 |
| 37 | 18808.43539 | 17674.99412 | 11158.7375 | 0 | 0 | 0 | 0 |
| 38 | 18789.47032 | 17637.98907 | 11018.01896 | 0 | 0 | 0 | 0 |
| 39 | 18772.24795 | 17604.38443 | 10890.23114 | 0 | 0 | 0 | 0 |
| 40 | 18756.64534 | 17573.94029 | 10774.46172 | 0 | 0 | 0 | 0 |
| 41 | 18742.54546 | 17546.4283 | 10669.84232 | 0 | 0 | 0 | 0 |
| 42 | 18729.83705 | 17521.63138 | 10575.54746 | 0 | 0 | 0 | 0 |
| 43 | 18718.41448 | 17499.34343 | 10490.79343 | 0 | 0 | 0 | 0 |
| 44 | 18708.17763 | 17479.36906 | 10414.83721 | 0 | 0 | 0 | 0 |
| 45 | 18699.03168 | 17461.5233 | 10346.97542 | 0 | 0 | 0 | 0 |
| 46 | 18690.88704 | 17445.63131 | 10286.5432 | 0 | 0 | 0 | 0 |
| 47 | 18683.65915 | 17431.52809 | 10232.91312 | 0 | 0 | 0 | 0 |
| 48 | 18677.26834 | 17419.05821 | 10185.49412 | 0 | 0 | 0 | 0 |
| 49 | 18671.63971 | 17408.07552 | 10143.73042 | 0 | 0 | 0 | 0 |
| 50 | 18666.70298 | 17398.44285 | 10107.10042 | 0 | 0 | 0 | 0 |
| 51 | 18662.39228 | 17390.03174 | 10075.11562 | 0 | 0 | 0 | 0 |
| 52 | 18658.64612 | 17382.72215 | 10047.31955 | 0 | 0 | 0 | 0 |
| 53 | 18655.40713 | 17376.40217 | 10023.28667 | 0 | 0 | 0 | 0 |
| 54 | 18652.62199 | 17370.96774 | 10002.62127 | 0 | 0 | 0 | 0 |
| 55 | 18650.24125 | 17366.32238 | 9984.95642 | 0 | 0 | 0 | 0 |
| 56 | 18648.21917 | 17362.37686 | 9969.95286 | 0 | 0 | 0 | 0 |
| 57 | 18646.51362 | 17359.04897 | 9957.29792 | 0 | 0 | 0 | 0 |
| 58 | 18645.08591 | 17356.26317 | 9946.70444 | 0 | 0 | 0 | 0 |
| 59 | 18643.90061 | 17353.95039 | 9937.90967 | 0 | 0 | 0 | 0 |
| 60 | 18642.92546 | 17352.04767 | 9930.67421 | 0 | 0 | 0 | 0 |
| 61 | 18642.1312 | 17350.49789 | 9924.78089 | 0 | 0 | 0 | 0 |
| 62 | 18641.49141 | 17349.24952 | 9920.03373 | 0 | 0 | 0 | 0 |
| 63 | 18640.98238 | 17348.25629 | 9916.2568 | 0 | 0 | 0 | 0 |
| 64 | 18640.58297 | 17347.47695 | 9913.2932 | 0 | 0 | 0 | 0 |
| 65 | 18640.27443 | 17346.87492 | 9911.00389 | 0 | 0 | 0 | 0 |
| 66 | 18640.04031 | 17346.41809 | 9909.2667 | 0 | 0 | 0 | 0 |
| 67 | 18639.86624 | 17346.07845 | 9907.97516 | 0 | 0 | 0 | 0 |
| 68 | 18639.73987 | 17345.83187 | 9907.03748 | 0 | 0 | 0 | 0 |
| 69 | 18639.65064 | 17345.65777 | 9906.37542 | 0 | 0 | 0 | 0 |
| 70 | 18639.5897 | 17345.53885 | 9905.92323 | 0 | 0 | 0 | 0 |
| 71 | 18639.54971 | 17345.46084 | 9905.62656 | 0 | 0 | 0 | 0 |
| 72 | 18639.52475 | 17345.41213 | 9905.44135 | 0 | 0 | 0 | 0 |
| 73 | 18639.51012 | 17345.38358 | 9905.33279 | 0 | 0 | 0 | 0 |
| 74 | 18639.50222 | 17345.36817 | 9905.27419 | 0 | 0 | 0 | 0 |
| 75 | 18639.49842 | 17345.36074 | 9905.24594 | 0 | 0 | 0 | 0 |
| 76 | 18639.49686 | 17345.3577 | 9905.23436 | 0 | 0 | 0 | 0 |
| 77 | 18639.49636 | 17345.35674 | 9905.2307 | 0 | 0 | 0 | 0 |
| 78 | 18639.49626 | 17345.35655 | 9905.22998 | 0 | 0 | 0 | 0 |
| 79 | 18639.49626 | 17345.35653 | 9905.22993 | 0 | 0 | 0 | 0 |
| 80 | 18639.49626 | 17345.35653 | 9905.22993 | 0 | 0 | 0 | 0 |

**S4 Table.** **Negative pressure value in axial direction of gas drainage borehole** **under prevention technology**

| **Distance**  **(m)** | **Negative pressure in gas drainage borehole（Pa）** | | | | | |
| --- | --- | --- | --- | --- | --- | --- |
|  | **t=1d** | **t=2d** | **t=5d** | **t=8d** | **t=12d** | **t=15d** |
| 15 | 20000 | 20000 | 20000 | 20000 | 20000 | 20000 |
| 16 | 19934.83477 | 19923.97389 | 19912.27757 | 19906.90681 | 19905.75276 | 19905.36169 |
| 17 | 19873.6479 | 19852.58921 | 19829.91063 | 19819.49699 | 19817.25935 | 19816.50109 |
| 18 | 19816.25439 | 19785.63012 | 19752.65014 | 19737.50627 | 19734.25222 | 19733.14953 |
| 19 | 19762.47507 | 19722.88758 | 19680.2549 | 19660.67867 | 19656.47221 | 19655.04678 |
| 20 | 19712.13649 | 19664.15924 | 19612.49143 | 19588.76642 | 19583.66848 | 19581.94097 |
| 21 | 19665.07086 | 19609.24933 | 19549.13385 | 19521.5298 | 19515.59835 | 19513.58838 |
| 22 | 19621.1159 | 19557.96856 | 19489.96372 | 19458.73701 | 19452.02713 | 19449.75339 |
| 23 | 19580.11483 | 19510.13397 | 19434.76997 | 19400.16404 | 19392.72806 | 19390.20826 |
| 24 | 19541.9162 | 19465.56889 | 19383.34872 | 19345.59456 | 19337.4821 | 19334.73306 |
| 25 | 19506.37382 | 19424.10279 | 19335.50322 | 19294.81974 | 19286.07784 | 19283.1155 |
| 26 | 19473.3467 | 19385.57115 | 19291.04363 | 19247.63814 | 19238.31134 | 19235.15081 |
| 27 | 19442.69892 | 19349.8154 | 19249.787 | 19203.85559 | 19193.98604 | 19190.64158 |
| 28 | 19414.29953 | 19316.68279 | 19211.55706 | 19163.28505 | 19152.91255 | 19149.39766 |
| 29 | 19388.02251 | 19286.02626 | 19176.18415 | 19125.74644 | 19114.90859 | 19111.23601 |
| 30 | 19363.7466 | 19257.70437 | 19143.50504 | 19091.06658 | 19079.79881 | 19075.98054 |
| 31 | 19341.35529 | 19231.58117 | 19113.36288 | 19059.07898 | 19047.41467 | 19043.46203 |
| 32 | 19320.73664 | 19207.52607 | 19085.60701 | 19029.62377 | 19017.59431 | 19013.51794 |
| 33 | 19301.78326 | 19185.4138 | 19060.09285 | 19002.54751 | 18990.1824 | 18985.99228 |
| 34 | 19284.39218 | 19165.12421 | 19036.68178 | 18977.70312 | 18965.03002 | 18960.73553 |
| 35 | 19268.46478 | 19146.54224 | 19015.24104 | 18954.94968 | 18941.99451 | 18937.60445 |
| 36 | 19253.90664 | 19129.55775 | 18995.64356 | 18934.15235 | 18920.93936 | 18916.46193 |
| 37 | 19240.62754 | 19114.06547 | 18977.76785 | 18915.18221 | 18901.73405 | 18897.17693 |
| 38 | 19228.54129 | 19099.96483 | 18961.49789 | 18897.91612 | 18884.25393 | 18879.62427 |
| 39 | 19217.56564 | 19087.15992 | 18946.72298 | 18882.23663 | 18868.38006 | 18863.68454 |
| 40 | 19207.62225 | 19075.55929 | 18933.33765 | 18868.03179 | 18853.99913 | 18849.24394 |
| 41 | 19198.63654 | 19065.07596 | 18921.24149 | 18855.19505 | 18841.00325 | 18836.19414 |
| 42 | 19190.53759 | 19055.62719 | 18910.33906 | 18843.62513 | 18829.2899 | 18824.43218 |
| 43 | 19183.2581 | 19047.13445 | 18900.53975 | 18833.22585 | 18818.76171 | 18813.86031 |
| 44 | 19176.73425 | 19039.52329 | 18891.75764 | 18823.90607 | 18809.32639 | 18804.38584 |
| 45 | 19170.90563 | 19032.72323 | 18883.91142 | 18815.57947 | 18800.89657 | 18795.92104 |
| 46 | 19165.71513 | 19026.66765 | 18876.92421 | 18808.16447 | 18793.38965 | 18788.38297 |
| 47 | 19161.10886 | 19021.29367 | 18870.72347 | 18801.58409 | 18786.72769 | 18781.69337 |
| 48 | 19157.03606 | 19016.54207 | 18865.24086 | 18795.76581 | 18780.83728 | 18775.77852 |
| 49 | 19153.449 | 19012.35716 | 18860.41211 | 18790.64142 | 18775.64938 | 18770.56908 |
| 50 | 19150.30286 | 19008.68667 | 18856.17693 | 18786.14694 | 18771.09918 | 18766.00001 |
| 51 | 19147.5557 | 19005.48165 | 18852.47883 | 18782.22243 | 18767.12601 | 18762.01035 |
| 52 | 19145.16831 | 19002.69636 | 18849.26503 | 18778.81187 | 18763.67317 | 18758.54318 |
| 53 | 19143.10413 | 19000.28815 | 18846.48633 | 18775.86305 | 18760.68779 | 18755.54542 |
| 54 | 19141.32919 | 18998.21739 | 18844.09699 | 18773.32741 | 18758.12073 | 18752.9677 |
| 55 | 19139.81196 | 18996.44729 | 18842.05456 | 18771.15995 | 18755.92639 | 18750.76426 |
| 56 | 19138.52331 | 18994.94386 | 18840.31984 | 18769.31902 | 18754.06264 | 18748.89278 |
| 57 | 19137.43639 | 18993.67578 | 18838.85667 | 18767.76626 | 18752.49064 | 18747.31425 |
| 58 | 19136.52652 | 18992.61427 | 18837.63185 | 18766.46645 | 18751.17471 | 18745.99286 |
| 59 | 19135.77114 | 18991.73299 | 18836.61499 | 18765.38734 | 18750.08222 | 18744.89584 |
| 60 | 19135.14969 | 18991.00797 | 18835.77842 | 18764.49955 | 18749.18343 | 18743.99332 |
| 61 | 19134.64351 | 18990.41743 | 18835.09703 | 18763.77644 | 18748.45136 | 18743.25821 |
| 62 | 19134.23578 | 18989.94174 | 18834.54816 | 18763.19397 | 18747.86166 | 18742.66607 |
| 63 | 19133.91138 | 18989.56328 | 18834.11147 | 18762.73054 | 18747.39249 | 18742.19495 |
| 64 | 19133.65684 | 18989.26631 | 18833.76882 | 18762.36691 | 18747.02435 | 18741.82528 |
| 65 | 19133.46021 | 18989.03691 | 18833.50413 | 18762.08601 | 18746.73997 | 18741.53972 |
| 66 | 19133.311 | 18988.86284 | 18833.30327 | 18761.87286 | 18746.52418 | 18741.32303 |
| 67 | 19133.20007 | 18988.73342 | 18833.15394 | 18761.71439 | 18746.36374 | 18741.16193 |
| 68 | 19133.11954 | 18988.63946 | 18833.04553 | 18761.59934 | 18746.24726 | 18741.04497 |
| 69 | 19133.06267 | 18988.57312 | 18832.96898 | 18761.5181 | 18746.16502 | 18740.96239 |
| 70 | 19133.02383 | 18988.52781 | 18832.9167 | 18761.46262 | 18746.10885 | 18740.90598 |
| 71 | 19132.99835 | 18988.49808 | 18832.8824 | 18761.42622 | 18746.072 | 18740.86898 |
| 72 | 19132.98245 | 18988.47952 | 18832.86098 | 18761.40349 | 18746.04899 | 18740.84588 |
| 73 | 19132.97312 | 18988.46864 | 18832.84843 | 18761.39017 | 18746.03551 | 18740.83233 |
| 74 | 19132.96809 | 18988.46277 | 18832.84166 | 18761.38298 | 18746.02823 | 18740.82502 |
| 75 | 19132.96566 | 18988.45994 | 18832.83839 | 18761.37952 | 18746.02472 | 18740.8215 |
| 76 | 19132.96467 | 18988.45878 | 18832.83705 | 18761.3781 | 18746.02328 | 18740.82006 |
| 77 | 19132.96435 | 18988.45841 | 18832.83663 | 18761.37765 | 18746.02282 | 18740.8196 |
| 78 | 19132.96429 | 18988.45834 | 18832.83655 | 18761.37756 | 18746.02273 | 18740.81951 |
| 79 | 19132.96429 | 18988.45833 | 18832.83654 | 18761.37755 | 18746.02273 | 18740.8195 |
| 80 | 19132.96429 | 18988.45833 | 18832.83654 | 18761.37755 | 18746.02273 | 18740.8195 |

**S5 Table. Negative pressure loss value of traditional drainage technology and Prevention Technology**

| **Time (d)** | **Negative pressure loss (kPa)** | **Time (d)** | **Negative pressure loss (kPa)** |
| --- | --- | --- | --- |
| 0 | 0 | 0 | 0 |
| 1 | 1.36 | 1 | 0.87 |
| 2 | 3.2 | 2 | 1.01 |
| 5 | 10 | 5 | 1.17 |
| 10 | 20 | 8 | 1.24 |
| 15 | 20 | 12 | 1.25 |
| 20 | 20 | 15 | 1.26 |
| 25 | 20 | 20 | 1.26 |
|  |  | 25 | 1.26 |

**S6 Table. Monitoring data of the average gas drainage concentration and gas flow**

| **Time**  **(d)** | **gas drainage concentration (%)** | | **gas flow (m^3^/min)** | |
| --- | --- | --- | --- | --- |
|  | **The prevention technology** | **Traditional drainage technology** | **The prevention technology** | **Traditional drainage technology** |
| 0 | 95 | 76 | 0.0055 | 0.0054 |
| 3 | 85 | 71 | 0.0054 | 0.0052 |
| 6 | 85 | 63 | 0.0053 | 0.0049 |
| 9 | 84 | 52 | 0.0053 | 0.0048 |
| 12 | 81 | 45 | 0.0051 | 0.0042 |
| 16 | 79 | 43 | 0.0050 | 0.0038 |
| 20 | 75 | 42 | 0.0050 | 0.0038 |
| 24 | 70 | 41 | 0.0048 | 0.0029 |
| 27 | 69 | 40 | 0.0047 | 0.0030 |
| 30 | 68 | 39 | 0.0046 | 0.0030 |
| 33 | 66 | 31 | 0.0042 | 0.0029 |
| 36 | 64 | 29 | 0.0040 | 0.0032 |
| 39 | 63 | 24 | 0.0038 | 0.0028 |
| 43 | 64 | 27 | 0.0038 | 0.0029 |
| 46 | 62 | 27 | 0.0038 | 0.0027 |
| 50 | 63 | 24 | 0.0036 | 0.0026 |
| 53 | 61 | 26 | 0.0034 | 0.0024 |
| 56 | 59 | 26 | 0.0034 | 0.0024 |
| 59 | 61 | 24 | 0.0032 | 0.0022 |
| 63 | 59 | 23 | 0.0032 | 0.0023 |
| 66 | 61 | 22 | 0.0032 | 0.0019 |
| 69 | 57 | 22 | 0.0030 | 0.0021 |
| 72 | 56 | 19 | 0.0029 | 0.0022 |
| 75 | 56 | 17 | 0.0029 | 0.0019 |
| 79 | 53 | 18 | 0.0029 | 0.0019 |
| 82 | 58 | 20 | 0.0029 | 0.0018 |
| 86 | 58 | 18 | 0.0029 | 0.0017 |
| 90 | 57 | 18 | 0.0029 | 0.0017 |
